# Phylogenetic and functional analysis of tiller angle control homeologs in allotetraploid cotton

**DOI:** 10.3389/fpls.2023.1320638

**Published:** 2024-01-31

**Authors:** Foster Kangben, Sonika Kumar, Zhigang Li, Avinash Sreedasyam, Chris Dardick, Don Jones, Christopher A. Saski

**Affiliations:** ^1^ Department of Plant and Environmental Sciences, Clemson University, Clemson, SC, United States; ^2^ Genome Sequencing Center, HudsonAlpha Institute for Biotechnology, Huntsville, AL, United States; ^3^ United States Department of Agriculture-Agricultural Research Service (USDA-ARS), Appalachian Fruit Research Station, Kearneysville, WV, United States; ^4^ Department of Agricultural Research, Cotton Incorporated, Cary, NC, United States

**Keywords:** branch angle, CRISPR, plant architecture, petiole angle, tac1, upland cotton

## Abstract

**Introduction:**

Plants can adapt their growth to optimize light capture in competitive environments, with branch angle being a crucial factor influencing plant phenotype and physiology. Decreased branch angles in cereal crops have been shown to enhance productivity in high-density plantings. The Tiller Angle Control (TAC1) gene, known for regulating tiller inclination in rice and corn, has been found to control branch angle in eudicots. Manipulating TAC1 in field crops like cotton offers the potential for improving crop productivity.

**Methods:**

Using a homolog-based methodology, we examined the distribution of TAC1-related genes in cotton compared to other angiosperms. Furthermore, tissue-specific qPCR analysis unveiled distinct expression patterns of TAC1 genes in various cotton tissues. To silence highly expressed specific TAC1 homeologs in the stem, we applied CRISPR-Cas9 gene editing and Agrobacterium-mediated transformation, followed by genotyping and subsequent phenotypic validation of the mutants.

**Results:**

Gene duplication events of TAC1 specific to the Gossypium lineage were identified, with 3 copies in diploid progenitors and 6 copies in allotetraploid cottons. Sequence analysis of the TAC1 homeologs in Gossypium hirsutum revealed divergence from other angiosperms with 1-2 copies, suggesting possible neo- or sub-functionalization for the duplicated copies. These TAC1 homeologs exhibited distinct gene expression patterns in various tissues over developmental time, with elevated expression of A11G109300 and D11G112200, specifically in flowers and stems, respectively. CRISPR-mediated loss of these TAC1 homeologous genes resulted in a reduction in branch angle and altered petiole angles, and a 5 to 10-fold reduction in TAC1 expression in the mutants, confirming their role in controlling branch and petiole angles. This research provides a promising strategy for genetically engineering branch and petiole angles in commercial cotton varieties, potentially leading to increased productivity.

## Highlight

The Tiller Angle Control (*TAC1*) gene is duplicated in the *Gossipum* lineage and regulates branch angle with significant A-subgenome expression bias. Manipulating this gene in Upland cotton can potentially improve productivity through high-density planting.

## Introduction

Improving crop performance involves various factors such as optimized light capture, carbon assimilation, and photosynthetic efficiency to achieve higher yield (Kant et al., 2012; Murchie and Burgess, 2022). The precise manipulation of plant architecture, particularly branching, can significantly enhance crop management and productivity. By controlling branching and limiting plant-to-plant interference, farmers can increase planting density, photosynthesis and carbon assimilation, boost yield, and improve mechanization efficiency (McGarry et al., 2016; Fladung, 2021).

Plant architecture is defined by the spatial configuration and morphological traits of its aerial and rooting structures in three dimensions. This fundamental feature has undergone modification throughout crop domestication and is crucial to the plant’s adaptability and productivity (Yang and Hwa, 2008; Cai et al., 2016). Plant architecture is the outcome of an intricate interplay between diverse regulatory mechanisms encompassing genetic programming, various hormone signaling pathways, and response to environmental cues such as light, gravity, and mechanical forces. A plant’s form, stem and leaf arrangement, and overall growth patterns are all influenced by these factors (Reinhardt and Kuhlemeier, 2002; Li et al., 2022; Sun et al., 2022). In angiosperms, variations in branch angles and other structures, such as leaves and branches, are viewed as adaptive strategies in modulating light interception efficiency. The ability to intercept light under varying conditions can also affect plant growth patterns and structures (Duursma et al., 2012). A key aspect of the success of the Green Revolution was the genetic modification of cereal crops, such as wheat, to improve their productivity and growth potential through breeding and selection for short, robust stems. This led to the development of wheat varieties that could withstand damage from wind and rain (Peng et al., 1999; Reinhardt and Kuhlemeier, 2002; Song and Zhang, 2009). Likewise, the architecture and yield of corn has been substantially improved through selection for reduced tillering, upright leaf angles, and increased apical dominance compared to its ancestor, teosinte (Whipple et al., 2011; Schmidt et al., 2016). Modern breeding has focused on the development of elite cultivars with erect, compact stem architecture, upright leaf angles, reduced branching, high harvest indices, and increased seed yield (Li et al., 2013). It is now commonly recognized that branches oriented vertically near the meristem and more horizontally at the lower canopy, are considered ideal for optimal light interception and correlated with higher yields (Kaggwa-Asiimwe et al., 2013; Sun et al., 2022). Using modern tools, plant architecture, including branch and leaf orientation, can now be manipulated with genome editing tools such as CRISPR to optimize light exposure and water uptake.

The Tiller Angle Control 1 (*TAC1*) gene has been identified as a crucial regulator of upright lateral organ orientation in rice, corn, and rapeseed (Yu et al., 2007; Ku et al., 2011; Li et al., 2017). *TAC1* belongs to a small gene family, known as the IGT family, which controls the orientation of organ growth by inhibiting response to gravity in various plants, including grasses, dicots, and trees. Along with *TAC1*, the IGT family includes LAZY and DRO subfamilies that promote upward orientation of branches and downward growth of lateral roots by facilitating auxin redistribution (Duvick, 2005; Yu et al., 2007; Ku et al., 2011; Li et al., 2017). Although LAZY and DRO mechanisms have been extensively studied, little is known about *TAC1*, which has been suggested as a negative regulator of LAZY (Hollender et al., 2020). Model species such as Arabidopsis and rice have a single copy of the *TAC1* gene (Guseman et al., 2017; Hollender et al., 2020). The loss of *TAC1* function leads to a broom or pillar-like plant architecture, characterized by vertically oriented branches, leaves, inflorescence, flower buds, and tillers (Dardick et al., 2013). Under optimal light conditions the expression of *TAC1* is up-regulated, potentially via the constitutive photomorphogenesis (*COP*1) gene, resulting in wider branch angles that enhance photoreception efficiency (Waite and Dardick, 2018).

Plants exhibiting loss of *TAC1* phenotypes occupy less space which allows for a higher planting density corresponding to an increased yield. Loss of function of *TAC1* results in a more inclined orientation of branch growth when plants are grown in prolonged darkness (Waite and Dardick, 2018; Hollender et al., 2020). In *Arabidopsis* and peach *TAC1* is primarily expressed in the apical shoots and upper sections of the stem, as well as the upper laterals. *TAC1* expression in rice is temporal peaking at 60 days after sowing and declining to a minimum at 100 days after sowing, especially during the heading stage. This leads to a decrease in leaf shading and increases photosynthetic efficiency (Yu et al., 2007; Dardick et al., 2013).

Cotton is a widely grown crop, covering 5% of the world’s farmland and valued for its fiber, seeds, and oil. It’s vital to the U.S. economy, which is the top cotton exporter and ranks third in production after China and India (Wang et al., 2012; Yuan et al., 2015; Shahbandeh, 2021). One approach to maximizing cotton yield is through the augmentation of planting densities (Kaggwa-Asiimwe et al., 2013; Khan et al., 2020). Current recommendations based on elite cotton varieties range from 20,000 to 45,000 cotton plants per acre (ppa). Plant populations exceeding 60,000 or falling below 20,000 ppa can result in management challenges and significant reductions in yield potential (Adams et al., 2019). Conversely, leading cotton seed suppliers often advocate for a plant density of 50,000 plants ha-1 to achieve optimal yields per hectare, resulting in a range of 700 to 1000 kg/ha. Higher plant density per unit area confers numerous advantages, including increased yield due to a greater number of plants within a given space, efficient utilization of resources such as water, nutrients, and sunlight, decreased weed growth and soil erosion, improved pest management, and enhanced microclimate conditions (Kaggwa-Asiimwe et al., 2013; Adams et al., 2019; Basu and Parida, 2021). However, under poor soil and seed conditions, higher planting population densities of up to 129,000 plants ha-1 have been reported to achieve similar yields (Norton et al., 1995; Fok, 1998). The expected cotton yield is contingent upon various factors such as the specific cotton variety cultivated, land availability, soil type, climatic conditions, planting density, available resources, nutrition, and management practices.

One possible explanation for the limited increase in yield observed in high-density cotton plantings could be linked to the prevalent spreading or horizontally oriented branching patterns seen in most cotton cultivars. In fact, small acreage farmers opt for crop varieties with narrower branch angles that can support higher plant density per area while also facilitating mechanical field management. Cotton plants exhibit two types of branches: monopodial and sympodial. Monopodial branches, also known as vegetative branches, originate from the main stem and continue to grow indefinitely throughout the plant’s development. Monopodial branches primarily grow in a nearly upright position. In contrast, sympodial branches, or fruiting branches, are determinate and bear the inflorescence, which eventually develops into the cotton bolls. Sympodial branches typically grow almost laterally from the main stem and are largely responsible for bearing the reproductive buds (squares). Fruiting branch angles ranging from 36.86° to 64.56° have been reported (Shao et al., 2022). With multiple branch types, cotton plants can allocate resources efficiently to both vegetative growth and reproduction (Gore, 1935; McGarry et al., 2016). Altered branch orientation for both branches could potentially impact cotton plant architecture. Given the desire for cotton varieties with narrow branch angles, it is critical that we improve our understanding of how monopodial and sympodial branch angles are regulated and identify strategies to develop improved germplasm with branch angles better suited for very high planting densities.

This study aimed to analyze the gene content and variation of *TAC1* in the genomes of diploid and allotetraploid cottons as well as other Angiosperms. Our goals were to: (I) identify *TAC1* orthologs in cotton species and other plant species; (II) determine homeologous gene expression profiles of *TAC1* in various tissues (stem, leaf, flower, fiber, meristem and root) in the allotetraploid cotton genotype Coker312; (III) functionally profile via CRISPR/CAS9 knockout the homeologous *TAC1* copies that are the most expressed in stem tissue; and (IV) develop gene editing strategies to modify cotton branch angles.

## Materials and methods

### Sequence alignment and phylogenetic analysis

To uncover potential *TAC1*-related genes we conducted a blast search and motif analysis of the Ppe*TAC1* coding sequence from *Prunus persica* in the full genome sequences of *Gossypium* species (*G*. *hirsutum*, *G*.*raimondii*, *G*. *arboreum*, *G*. *barbadense*, *G*. *darwinii*, *G*.*tomentosum*) and other selected plants. For *G. hirsutum* we used the widely used and well-annotated Coker312 genotype as it is known for its regenerative capacity (Kumar et al., 2021). Coding and protein sequences were obtained from Phytozome, and the BLOSUM62 matrix was used in the blast search with an expected value of 1e-5. Amino acid sequences were concatenated for species with multiple copies using the concatenate feature in Geneious Prime software version 2023.2.1. Multiple alignments were performed with MUSCLE v5.1 (Edgar, 2004), and phylogenetic trees were constructed using the RAxML GAMMA GTR and the Neighbor-Joining method with 5,000 bootstrap replicates (Stamatakis, 2014; Kozlov et al., 2019). Genomic data for the analysis of most of the crops was retrieved from Phytozome Genome Database (Goodstein et al., 2012)

### Total RNA isolation

Samples of tissue were collected 14 days after anthesis (dpa) and immediately frozen. The tissue was then ground into a fine powder using a pestle and mortar in liquid nitrogen. Total RNA was extracted from 100mg of the tissue from leaf (basal, midsection and apical part), stem (between the 5^th^ and 15^th^ node and sliced into smaller pieces), meristem (apical part at 30cm downward), root (bulk roots and root tips and homogenized), flower (day before bloom), and fiber (14dpa) of the Coker 312 cotton genotype using a modified CTAB protocol method (Kumar et al., 2021). The purity and concentration of the extracted RNA were measured using a Nanodrop 8000 UV-Vis Spectrophotometer (Thermo Scientific).

### Quantitative real-time PCR

Six differentially expressed *TAC1* genes (GhCoker.A08G143500, GhCoker.D08G158700, GhCoker.A11G109300, GhCoker.D11G112200, GhCoker.A12G131200 and GhCoker.D12G137700) and three biological replicates each of leaf, stem, meristem, root, flower and fiber of Coker312 were used for qPCR. For mutants three biological replicates of stem tissue were used for qPCR analysis. For cDNA synthesis 1 ug of total RNAs was extracted from leaf, stem, meristem, root, flower, and fiber. The first strand of cDNA was synthesized using the M-MuLV reverse transcriptase (New England Biolabs, USA) and primed by d(T)25-VN following the manufacturer’s instructions. qPCR of gene transcripts was carried out using an iCycler iQ system (Bio-Rad, Hercules, CA, USA) in 20µL of PCR reaction solution using the Luna Universal qPCR Master Mix, New England Biolabs, USA, the SYBR^®^Green method was used for running the qPCR. Conditions for thermal cycling included initial denaturation at 95°C for 120 s, followed by 45 cycles of 95°C for 20 s, 60°C 30 s, and 72°C for 20s. Lastly a unique melting curve was performed from 60.0°C to 95.0°C in 0.5°C increments to amplify distinctive PCR product. One reference gene, *GhPP2A1* was used for normalization of the expression data. The reference gene was chosen because of its uniform expression between cells of different tissues and under variable experimental conditions (Artico et al., 2010). The Ct values of four technical samples for each of the three biological replications were used to estimate the relative expression of genes using the 2^−ΔΔCt^ equation (Schmittgen and Livak, 2008). Except for the reference genes, specific primer pairs were designed from conserved coding sequence and listed in **Supplementary Table S1**.

### Dual guide RNA design, transformation, validation of homeologous genome edits, and prediction of off-target site editing

The modified binary vector *pCSbar* (a *bar* gene cassette was cloned into *Eco*RI-*Hin*dIIIsites of pAMBIA1300) integrating *spCAS9* genes and two sgRNAs cassettes were prepared for cotton transformation (**Supplementary Figure S1**). The *spCAS9* driven by a dual 35S promoter was cloned into *Eco*RI site of *pCSbar*, and then the two synthesized sgRNAs (Synbio, NJ, USA) were integrated into the *Avr*II site of *pCSbar-Cas9*, and the result was in the final construct. The two crRNAs targeted the flanking sequences of a 128-base pair region specific to the second exon of the GhCoker.D11G112200 and GhCoker.A11G109300 driven by AtU6-26t and AtU6-29t promoters, respectively. PAM sequences are highlighted. The two crRNAs share 100% similarities to conserved regions of the two homologs GhCoker.D11G112200 and GhCoker.A11G109300 but low similarities to the other four homeologs of GhCoker.A08G143500.1, GhCoker.D08G158700.1, GhCoker.D12G137700.1 and GhCoker.A12G131200.1 (**Supplementary Table S2**).

The *Agrobacterium* (EHA105) mediated transformation and plant regeneration protocol was adopted from a standard transformation publication (Jin et al., 2012). The regenerated plants were transplanted into one-gallon pots (Dillen Products, Middlefield, OH) containing commercial potting mixture soil (Fafard 3-B Mix, Fafard Inc., Anderson, SC, USA) and were developed under the greenhouse system conditions (Biosystems Research Complex, Clemson University) for morphological observation and materials harvesting. Three independent transgenic events (*tac1-72*, *-73* and *-74*, verified by PCR of *bar* and *Cas9*) showing the phenotype were selected to verify the genotype. The total DNA obtained from each was subjected to PCR. The primer pair: F-AGATGGGCTTGCACGAAATGTTAAG and R-CGTTTTTGGCAGGAAGAGRAGATG were carefully selected to amplify the region overlapping the target sequences of the two crRNAs in the two homologous. The PCR products were cloned into pGEM-T-Easy vector and sequenced using standard Sanger sequencing techniques. The sequencing was conducted at the Genomics Core, Biosciences at Arizona State University. The chromatograms and sequence analysis were conducted in Geneious Prime (**Supplementary Figure S2**, **Supplementary Table S3**). To confirm the *tac1*-73 knockout genotype, isolated gDNA was sequenced at Hudson Alpha Institute of Biotechnology, 2x150bp paired-end reads at an average coverage of 12.58X across the genome. Reads were trimmed using Trimmomatic (Bolger et al., 2014) to remove Illumina adaptor sequences, and aligned to the *G. hirsutum* ‘Coker312’ genome version 1.1 (*Gossypium hirsutum* Coker v1.1, DOE-JGI, http://phytozome.jgi.doe.gov/info/GhirsutumCoker_v1_1) using Burrow-Wheeler Alignment (bwa-mem) (Li, 2013). The aligned BAM file for the *tac1*-73 knockout sample was investigated using Integrative Genomics Viewer (IGV) to verify the Crispr-Cas9 target sites (Robinson et al., 2011). BAM files were prepared for genotyping using samtools to eliminate duplicate reads (Danecek et al., 2021). Off-target single nucleotide polymorphisms were genotyped using samtools integrated with Varscan2 mpileup2snp (Koboldt et al., 2012; Danecek et al., 2021). Investigation of possible off-target indels or large structural variants was performed with Varscan2 mpileup2indel and Delly’s structural variant caller (Koboldt et al., 2012; Rausch et al., 2012; Danecek et al., 2021). Guide RNAs were aligned to the Coker312 genome with BLASTN (wordsize=7) and only found 8 hits with identity, **Supplementary Table S4**. Bedtools (Quinlan and Hall, 2010) intersect was used to determine that no variants were found in these regions when intersected with the variant call file (.VCF), data not shown.

### Phenotyping angle measurements

Branch and petiole angles were measured manually with a protractor and digitally in Adobe Photoshop to estimate the angle between the main stem to the branch and petiole (BioRender.com, 2023), **Supplementary Figure S3** (Sun et al., 2016a). Data was collected from five representative sympodial branches and two monopodial branches per plant and computed mean branch and petiole angles similar to (Li et al., 2017). We also collected data on branch length, plant height, boll count. Data was collected from the T_0_ mutants and wild type Coker 312 plants.

### Screening and verification of mutants

Stable integration of the CRISPR/Cas9 T-DNA was determined by screening the T_1_ mutants using the *BAR* gene selectable marker. The *BAR* gene has been extensively utilized and researched as a positive selectable marker for herbicide resistance, providing plants with resistance to phosphinothricin (PPT), the active ingredient in the broad-spectrum herbicide known as Basta. This functionality facilitates the elimination of non-transformed individuals and the selective advancement of transformants in plant regeneration processes (Thompson et al., 1987; Rathore et al., 1993; Hahn et al., 2017).

### Statistical analysis

The analysis of variance was performed using JMP Pro version 16.1 (SAS Institute, Cary, NC, USA). Mean separation was assessed through a one-way ANOVA with Tukey’s HSD test (p<0.05).

## Results

### Phylogenetic analysis of *TAC1* in the angiosperms

**Figure 1:**
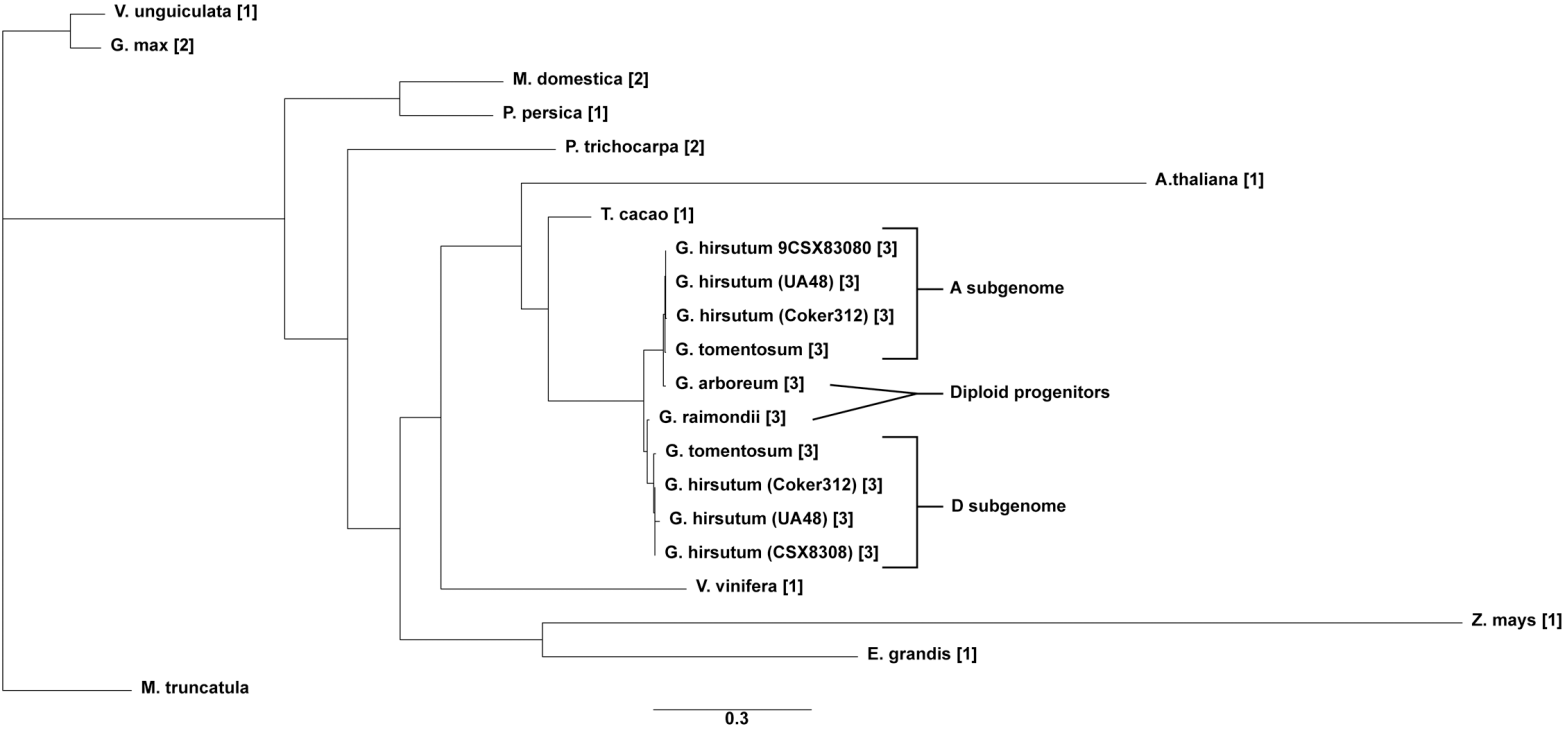
The phylogenetic tree of the TAC1 gene in cotton and other plant species. The tree was constructed by RAxML GAMMA GTR using the Neighbor-Joining method with 5000 bootstrap replicates (Stamatakis, 2014; Kozlov et al., 2019). The bar indicates an evolutionary distance of 2.0%.

Copy number and phylogenetic analysis was conducted to examine the distribution of cotton *TAC1* orthologs in a range of representative angiosperms, encompassing both species with known single copies (e.g. *Prunus persica, Zea mays, Vitus vinifera, Vigna unguiculata, Theobroma cacao, Medicago trunculata*, and *Eucalyptus grandis*) and those with two copies (*Glycine max* and *Populus trichocarpa*), **Table 1**. Analysis revealed that *TAC1* gene copy number in *Medicago truncatula*, a basal eudicot species, is most divergent from *V. unguiculata* in the phylogenetic tree (**Figure 1**). Our findings revealed a separate clade for the Rosaceae family members, *Prunus persica* (1 copy) and *Malus domestica* (2 copies), respectively. The analysis also showed a separate clade for the Fabaceae family member *V*. *unguiculata* and *G*. *max* both with 1 copy each. Notably, we detected an additional *TAC1* copy in the lineage containing the diploid cotton progenitor species *G. arboreum* and *G. raimondii*, each possessing three *TAC1* copies, indicating a *TAC1* duplication event specific to this lineage (**Figure 1**). As anticipated, within the *Gossypium* clade, the *TAC1* gene copy number remained consistent, with three copies in both the A and D subgenomes in all examined allotetraploid cotton species (*G. hirsutum* and *G. tomentosum*) examined (**Figure 1**; **Table 2**). As expected, the A-subgenome diploid progenitor, *Gossypium arboreum*, and the D-subgenome diploid progenitor, *Gossypium raimondii*, are ancestral **Table 1**; **Figure 1**. Furthermore, the phylogenetic analysis also revealed a close relationship between *T.cacao* (cocoa) and the clade containing the cotton lineages highlighting the relationship of both *Theobroma cacao* and *Gossypium* spp. within the Malvaceae family.

**Table 1:** List of putative *TAC1* orthologs in cotton and other plant species.

Genus	Species	Ploidy	Copy number	Cotyledon	Reference
*Gossypium*	*Arboretum*	Diploid	3	Eudicot	(Li et al., 2015; Zhang et al., 2015)
*Gossypium*	*hirsutum (CSX8308)*	Allotetraploid	6	Eudicot	(Phytozome, 2022b)
*Gossypium*	*hirsutum (Coker 312)*	Allotetraploid	6	Eudicot	(Phytozome, 2022a)
*Gossypium*	*hirsutum (UA48)*	Allotetraploid	6	Eudicot	(Phytozome, 2022c)
*Gossypium*	*raimondii*	Diploid	3	Eudicot	(Paterson et al., 2012)
*Gossypium*	*tomentosum*	Allotetraploid	6	Eudicot	(Chen et al., 2020)
*Arabidopsis*	*thaliana*	Diploid	1	Eudicot	(Cheng et al., 2017)
*Prunus*	*persica*	Diploid	1	Eudicot	(Verde et al., 2013)
*Populus*	*trichocarpa*	Diploid	2	Eudicot	(Tuskan et al., 2006)
*Malus*	*domestica*	Diploid	2	Eudicot	(Daccord et al., 2017)
*Glycine*	*max*	Diploid	2	Eudicot	(Schmutz et al., 2010)
*Eucalyptus*	*grandis*	Diploid	1	Eudicot	(Myburg et al., 2014)
*Medicago*	*truncatula*	Diploid	1	Eudicot	(Tang et al., 2014)
*Theobroma*	*cacao*	Diploid	1	Eudicot	(Motamayor et al., 2013)
*Vigna*	*unguiculata*	Diploid	1	Eudicot	(Lonardi et al., 2019)
*Vitus*	*vinifera*	Diploid	1	Eudicot	(Jaillon et al., 2007)
*Zea*	*mays*	Diploid	1	Monocot	(Zhang et al., 2009))

**Table 2:** TAC1 gene distribution in cotton.

Genus_species (cultivar)	Gene name	Subgenome (A/D)	Chromosome	Paired
*G. hirsutum* (Coker312)	GhCoker.A12G131200.1	A	12	Yes
	GhCoker.D12G137700.1	D	12	
	GhCoker.A11G109300.1	A	11	Yes
	GhCoker.D11G112200.1	D	11	
	GhCoker.A08G143500.1	A	8	Yes
	GhCoker.D08G158700.1	D	8	
*G. hirsutum* (CSX8308)	GhCSX8308.A12G132100.1	A	12	Yes
	GhCSX8308.D12G133900.1	D	12	
	GhCSX8308.A11G111000.1	A	11	Yes
	GhCSX8308.D11G111700.1	D	11	
	GhCSX8308.A08G142400.1	A	8	Yes
	GhCSX8308.D08G156700.1	D	8	
*G. hirsutum* (UA48)	GhUA48.A12G135900.1	A	12	Yes
	GhUA48.D12G133200.1	D	12	
	GhUA48.A11G109300.1	A	11	Yes
	GhUA48.D11G112000.1	D	11	
	GhUA48.A08G145900.1	A	8	Yes
	GhUA48.D08G152400.1	D	8	
*G. arboreum*	Gar12G15480	A	12	N/A
	Gar11G10910	A	11	N/A
	Gar08G16700	A	8	N/A
*G. raimodii*	Gorai.008G127500.1	D	8	N/A
	Gorai.007G111200.1	D	7	N/A
	Gorai.004G150000.1	D	4	N/A
*G. tometosum*	Gotom.A12G133000.1	A	12	Yes
	Gotom.D12G139200.1	D	12	
	Gotom.A11G112900.1	A	11	Yes
	Gotom.A11G112900.1	D	11	
	Gotom.A08G147800.1	A	8	Yes
	Gotom.D08G162100.1			

N/A, Not applicable.

**Figure 2:**
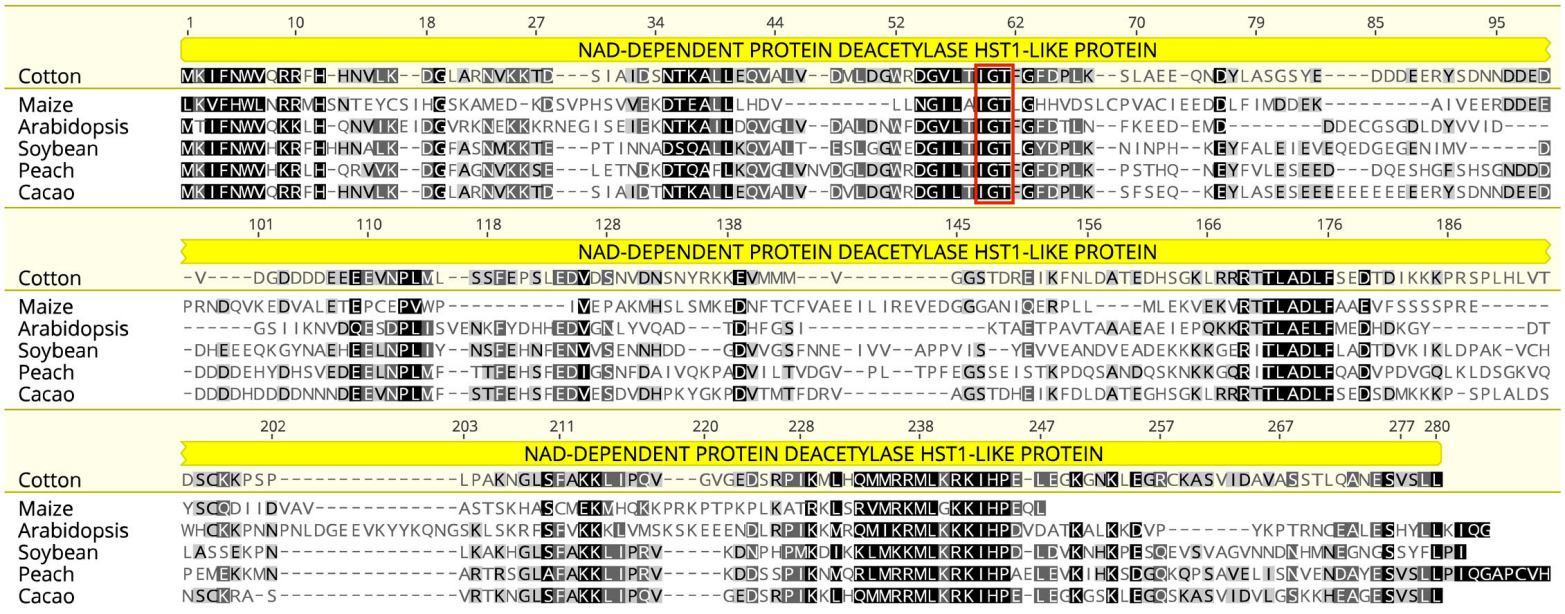
Multiple protein sequence alignment of predicted amino acid sequences of TAC1 orthologs cotton, peach, Arabidopsis, cacao, soybean, and maize. Highly conserved residues are highlighted in black with the IGT conserved domain indicated in red box at the 67-69bp position, which is typical of the IGT gene family.

**Figure 3:**
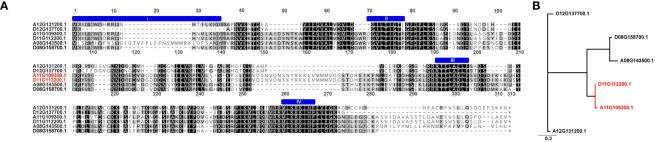
Multiple sequence alignment and dendrogram of TAC1 copies in (G) hirsutum (Coker312) and peach. **(A)** Six GhTAC1 protein sequences alignment. The previously reported 4 conserved domains (Dardick et al., 2013) are annotated by a blue “bar” above the sequence. **(B)** The homeologous copies on A12/D12 clade have the highest sequence identity to the single prunus copy. The six homologs within Coker312 are also quite diverged with only ~50% pairwise identity.

The *TAC1* gene is close to 300 amino acids in length and is predicted to contain an NAD-dependent protein deacetylase domain from the PantherDB (Mi and Thomas, 2009), **Figure 2**. A multiple sequence alignment of *TAC1* from cotton and various plant species is only modestly similar with an average percent identity of 33% and only 30 (9%) identical sites (**Figure 2**). Alignments revealed the presence of conserved domains of the *TAC1* gene in other plant species, such as the IGT conserved motif (**Figure 2**), which is known to have an impact on vertical shoot growth in a broad range of plant species (Roychoudhry and Kepinski, 2015; González-Arcos et al., 2019). Similarity, homeologs also differed significantly among the *TAC1* copies. For example, the A11/D11 homeologs had high similarity at 96.8% at the amino acid level, while the A12/D12 and A08/D08 homeologs were 73% identical (**Figure 3A**; **Supplementary Table S5**). The regions of conserved sequence previously reported (Dardick et al., 2013) are highlighted as four domains and are conserved among all the homeologs, **Figure 3A**. Examination of the *GhTAC1* phylogeny within the allotetraploid shows A08/D08 and A11/D11 homeologous gene pairs showed separate clades forming from the A12/D12 gene pairs (**Figure 3B**).

### Expression profiles of *GhTAC1* homeologs exhibit subgenome bias and vary by tissue type

**Figure 4:**
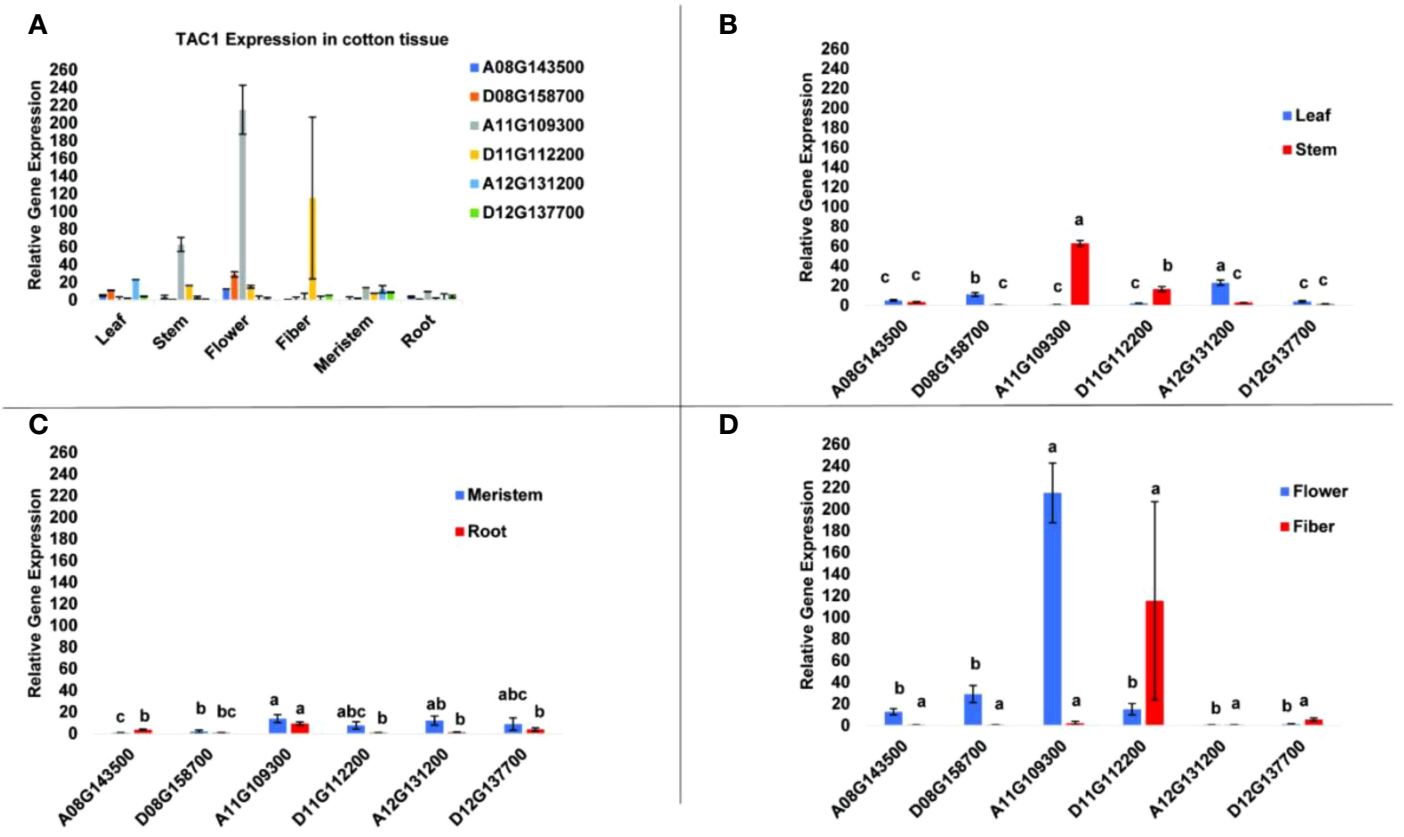
**(A)** Expression profiles of putative *TAC1* homologs in various cotton tissue. **(B)** leaf and stem, **(C)** meristem and root, **(D)** flower and fiber tissues. RT-qPCR was performed using template cDNA primed by Poly T_(25)_ from total RNA that was isolated from 100 mg of tissue. The statistical difference between groups was determined by one-way ANOVA with a *post-hoc* Tukey HSD Test. Means not sharing the same letter are statistically significantly different (P < 0.05). All the data presented as the mean ± SD (n=3).

To determine tissue-specific expression patterns of the six *GhTAC1* genes, we performed qPCR analysis in the Coker 312 cotton genotype at various stages of reproductive and vegetative growth utilizing gene and homoeolog-specific primers. Various *TAC1* homeologs displayed subgenome expression bias (A-subgenome dominance in all experiments) and differential expression in various cotton tissue sources (**Figure 4A**; **Table 3**). In vegetative tissues (leaf, stem, meristem, and roots) significant expression with subgenome bias was observed for the A11 *TAC1* homeolog (GhCoker.A11G109300) in stem tissue with over 10-fold expression compared to the other five homeologous copies (**Figure 4B**; **Table 3**). In leaf tissue GhCoker.A12G131200 was highly expressed compared to the other five homeologs with about a 2-fold expression compared to the second gene (GhCoker.D08G158700) that was highly expressed (**Figure 4B** and **Table 3**). Fairly low relative expression levels were observed in meristem and root tissue of all *GhTAC1* homeologous copies (**Figure 4C**; **Table 3**) which was expected, particularly in roots. Interestingly, in flower and fiber tissues, high expression levels were observed for GhCoker.A11G109300 (dominant copy) and GhCoker.D11G112200, respectively. The expression level of GhCokerA11.G109300 was over 7-fold higher in flower tissue and 20-fold higher in fiber compared to the other gene copies (**Figure 4D**; **Table 3**). The presence of multiple copies of the *GhTAC1* gene, along with its notably high expression in the flower tissue, may be the reason for the nearly horizontal growth pattern of the flower.

**Table 3:** Analysis of variance of TAC1 expression in multiple cotton tissues.

GENE	Leaf	Stem	Flower	Fiber	Meristem	Root
A08G143500	5.2a	3.5c	12.8b	1.0a	1.0a	3.8b
D08G158700	11.1b	1.0c	29.2b	1.0a	2.4a	1.0b
A11G109300	1.0c	63.0a	215.2a	2.4a	14.1a	9.5a
D11G112200	2.3c	16.6b	15.1b	115.4a	7.8a	1.0b
A12G131200	23.1a	3.0c	1.0b	1.0a	12.3a	1.4b
D12G137700	4.2a	1.3c	1.6b	5.6a	9.0a	4.1b
p-value (≤ 0.05)	<.0001	<.0001	<.0001	0.246	0.197	<.0001

Means not sharing the same letter are statistically significantly different (P < 0.05).

### Targeted A11/D11 homeologous genome edits with dual guide RNAs

**Figure 5:**
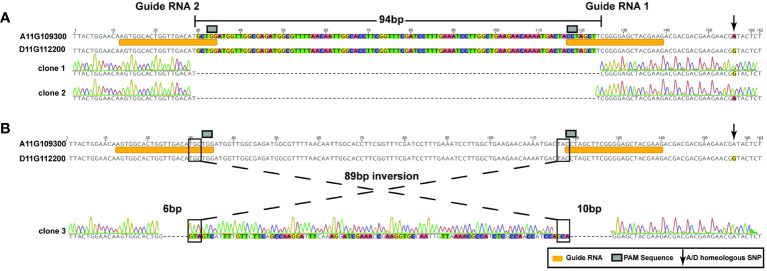
2 types of homeologous GhTAC1 edits by the CRISPR/Cas9 system in Coker312. **(A)** A 94-bp deletion that includes the PAM motif and a portion of the guide RNA. This deletion was found in the following events: tac1-72 (A and D subgenome) and in the A-subgenome of tac1-73 and tac1-74. **(B)** An 89bp inversion flanked by 6bp and 10bp deletions that include the PAM sequence and a portion of each guide RNA, respectively.

The dual guide RNAs used in this study to create a deletion were designed to target a 128-base pair (bp) region specific to the second exon of the GhCoker.D11G112200 and GhCoker.A11G109300 homeologous gene pair, **Supplementary Table S6**. Two types of mutations were observed at the target sites of SpCas9-edited lines that include a 94-base pair (bp) deletion (detected in most of the edited lines) and an 89bp inversion flanked by a 6bp and 10bp deletion, respectively, **Figures 5A, B**, **Supplementary Table S7**. The 94 bp deletion was found in both A and D subgenomes and included the PAM sequence, **Figure 5A**. In this event, the A and D subgenomes were clearly distinguished by numerous homeologous SNPs, arrow in **Figure 5A**. The 89 bp inversion flanked by 2 deletions that include approximately half of the gRNA1 and gRNA2 target sites was found only in the D-subgenome *TAC1* copy, (**Figure 5B**). The two edited lines with both 94bp deletions or a mosaic of the 94bp deletion and the 89bp inversion displayed the same branch/petiole angle inclination phenotype, **Supplementary Figure S3**.

### Genetic mutations and segregation analysis in T_1_ progeny

**Figure 6:**
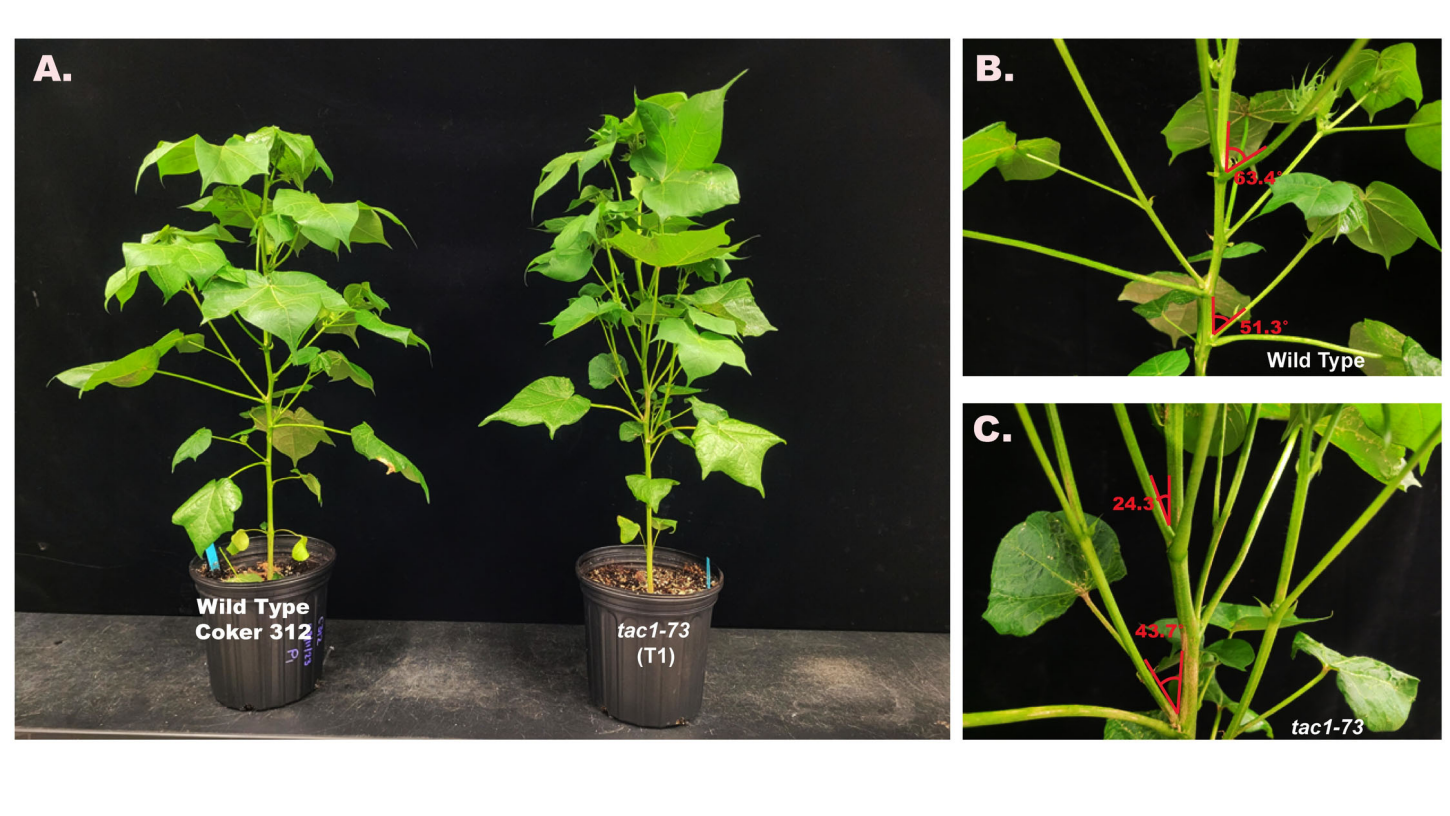
**(A)** Representative images depicting columnar phenotype inclination in wild type (Coker 312) *vs tac1-73* (T_1_ generation). **(B)** Branch angle measurements of wild type (Coker 312) and **(C)** Branch angle measurements and *tac1-73* (T_1_ generation).

The T_1_ seeds that were generated were sown to identify mutants and screen for individuals that may segregate with the genome editing reagents and the *TAC1* deletion. Among these mutants, *tac1*-73 and *tac1*-74 exhibited a favorable segregation ratio of 3:1, thus indicating a single copy (**Figure 6**). In our study, all 24 seeds of both *tac1*-73 and *tac1*-74 variants exhibited germination. Following screening for the presence of CAS9 in these mutants, 18 seedlings of *tac1*-73 and 17 of *tac1*-74 survived. These results verify that the knockout strategy for the mutants developed was successful and could be further validated by the phenotype observed five weeks after emergence (**Figure 6A**). The T_1_ plants of *tac1*-73 showed columnar branch formation as depicted in more inclined branch angles (**Figure 6B**) compared to wild type Coker 312 (**Figure 6C**).

### Validation of the expression profiles of *GhTAC1* homeologs in mutants

**Figure 7:**
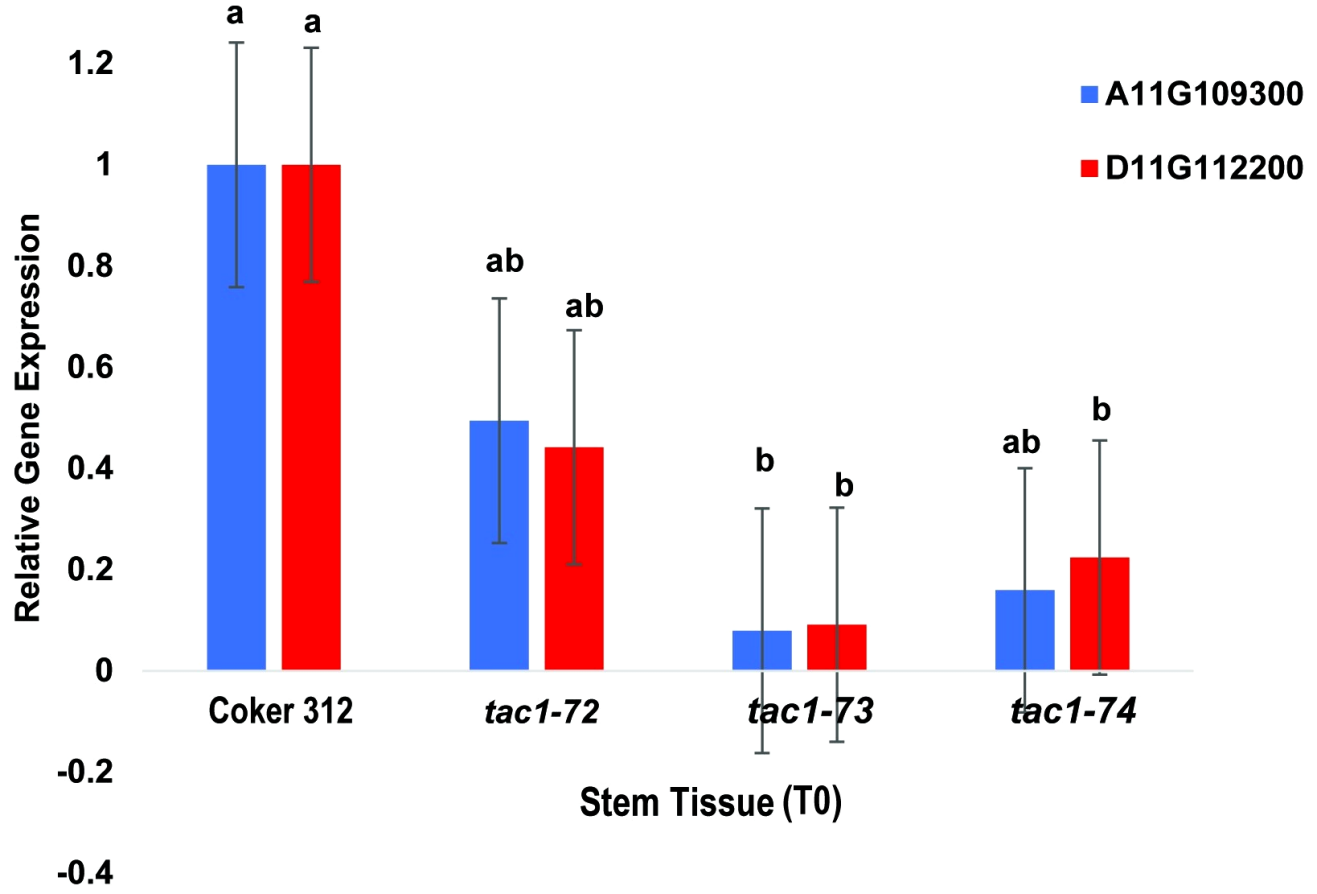
qPCR results of *GhTAC1* expression in stem tissue from wild type Coker 312 and *tac1-72*, *tac1-73*, and *tac1-74*. Error bars represent standard deviations of three biological replicates. Means not sharing the same letter are statistically significantly different (P < 0.05).

To assess the expression profiles of *TAC1* genes and specifically investigate the knockout effects on the homoeologous gene pairs GhCoker.A11G109300 and GhCoker.D11G112200 in *TAC1* mutant lines (*tac1*-72, *tac1*-73, and *tac1*-74) in comparison to the wild-type Coker 312, a quantitative polymerase chain reaction (qPCR) analysis was conducted using stem tissue samples. Homoeolog-specific primers were designed to target the 128bp knockout region within the second exon of GhCoker.A11G109300 and GhCoker.D11G112200 homoeologous gene pair. Remarkably, all three mutant lines exhibited significantly reduced expression levels of both GhCoker.A11G109300 and GhCoker.D11G112200 homoeologous gene pairs (**Figure 7**). Notably, when compared to the wild-type Coker 312, the *tac1*-73 and *tac1*-74 mutants displayed very low expression, with reductions of approximately 10-fold and 5-fold in *tac1*-73 and *tac1*-74 mutants, respectively. However, the *tac1*-72 mutant demonstrated a more moderate 2-fold reduction in expression. Based on the gene-specific expression patterns, GhCoker.A11G109300 (the dominant copy) exhibited lower expression levels in all three mutant plant lines when compared to GhCoker.D11G112200, except in *tac1*-72 where GhCoker.D11G112200 showed lower expression in comparison to GhCoker.A11G109300 (**Figure 7**).

### Alteration of A11/D11 *TAC1* expression leads to altered branch angle and impacts cotton morphology

**Figure 8:**
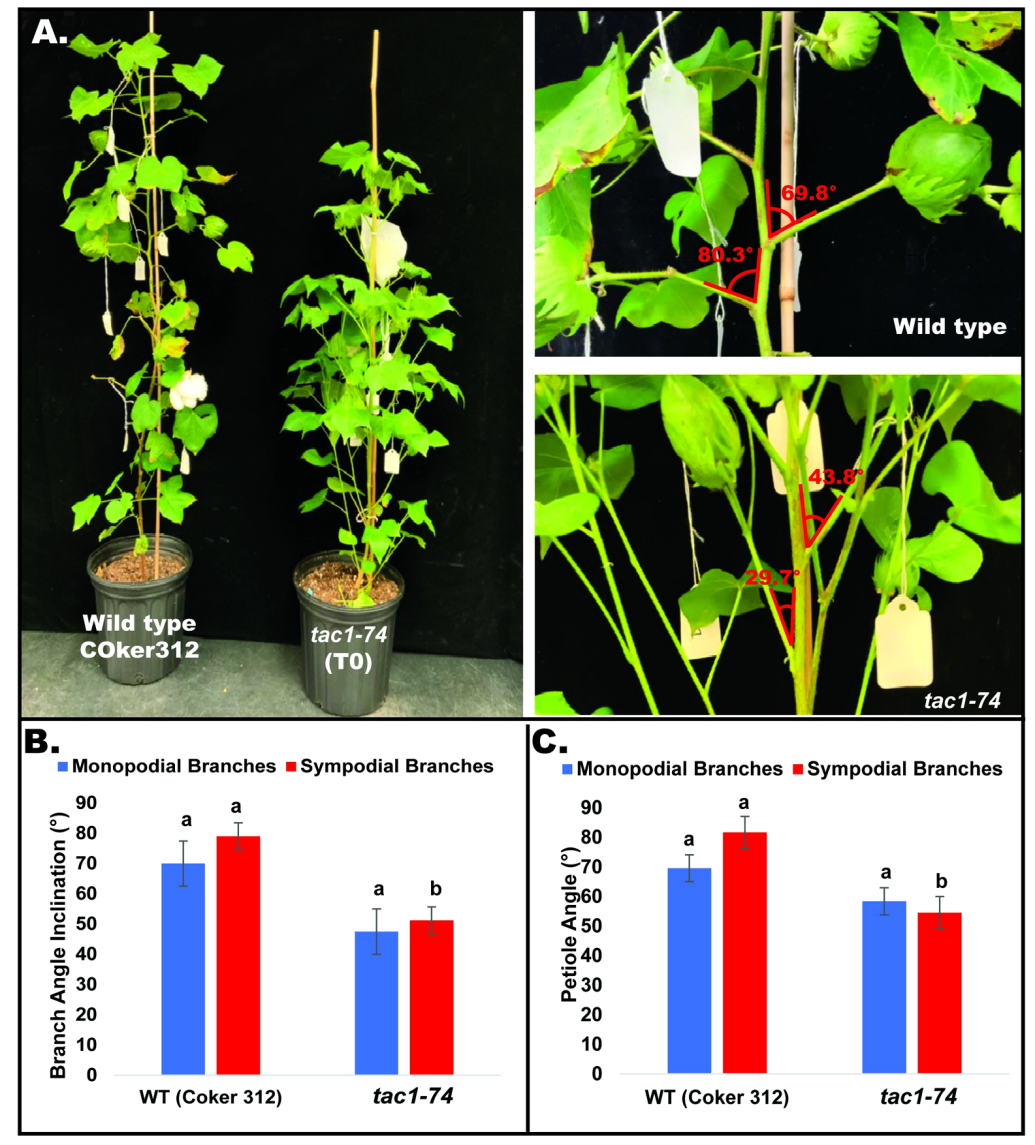
**(A)** Representative images depicting columnnar phenotype inclination in wild type (Coker 312) and mutant plant. **(B)** Mean branch angle measurements of wild type (Coker 312) and mutant plant (*tac1-74*). **(C)** Mean petiole angle measurements of wild type (Coker 312) and mutant plant (*tac1-74*). The statistical difference between groups was determined by one-way ANOVA with a *post-hoc* Tukey HSD Test. Means not sharing the same letter are statistically significantly different (P < 0.05). Errors bars represent SD.

To explore the role of *GhTAC1* in branch angle, the A11/D11 homoeologs were targeted because of their high levels of expression in stem tissue. Transgenic plants harboring targeted knockouts of the A11/D11 homoeologous gene pair were generated. Plants with the validated *GhTAC1* (A11/D11) knockouts (mutant) at the T_0_ stage displayed significant differences in branch inclination angles when compared to wild type Coker 312 plants (**Figure 8A**). Branch inclination angles were measured at flower bud formation. Mean branch inclination angles widely differed among the transgenic A11/D11 knockouts (transgenic positive plant) and wild type Coker 312 for both monopodial and sympodial branches (**Figures 8A–C**). The phenotyping results revealed significant differences in branch inclination angles among the different plant variants. For sympodial branches the mutant exhibited an angle of 51.2° while the wild-type Coker 312 measured 74.6°. On the other hand, no significant differences were observed in monopodial branch angles, with the mutant measuring 47.5° and the wild type Coker 312 being 57.5°. (see **Figure 8B**; **Table 4**). Regarding petiole angles, the mutant showed narrower angles in sympodial branches compared to wild type Coker 312. However, no significant difference was found in monopodial branches (see **Figure 8C**; **Table 5**). Furthermore, it was noted that the mutant plants displayed increased branching and leaf biomass in the basal region while displaying fewer branches and leaves in the apical region though not significantly different from the wild-type Coker 312 plant (**Supplementary Figures S4**-**S6**). To assess the impact of *GhTAC1* on cotton morphology, measurements were taken for boll count, plant height, and branch length. The analysis revealed no significant differences in these variables between the wild-type Coker 312 and the mutants (**Table 6**).

**Table 4:** Analysis of variance for branch angle inclination and petiole Angle.

Genotype	Mean Branch Angle		Mean Petiole Angle	
	Monopodial Branches	Sympodial Branches	Monopodial Branches	Sympodial Branches
Wild Type (Coker 312)	57.5a	74.6a	69.6a	81.7a
*tac1-74*	47.5a	51.2b	58.4a	54.6b
p-value (≤ 0.05)	0.3333	0.0075	0.1328	0.0011

Means not sharing the same letter are statistically significantly different (P < 0.05).

**Table 5:** Analysis of variance for TAC1 expression in stem tissue of wild type (Coker 312) and mutants (tac1-72 tac1-73 and tac1-74).

Primer	A11G109300	D11G112200
Coker 312	1.0a	1.0a
*tac1-72*	0.5ab	0.4ab
*tac1-73*	0.1b	0.1b
*tac1-74*	0.2ab	0.2b
p-value (≤0.05)	0.04	0.02

Means not sharing the same letter are statistically significantly different (P < 0.05).

**Table 6:** Analysis of variance for some morphological traits of wild type (Coker 312) and mutants (tac1-72 tac1-73 and tac1-74).

Genotype	Monopodial branch length	Sympodial branch length	Boll Count	Plant Height(cm)
Coker 312 (WT)	32.8a	16.3a	14a	90.4a
*tac1-72*	37.3a	15.0a	14a	100.8a
*tac1-73*	38.1a	15.2a	15a	102.9a
*tac1-74*	38.2a	15.0a	14a	101.6a
p-value (≤0.05)	0.154	0.901	0.983	0.223

Means not sharing the same letter are statistically significantly different (P < 0.05).

## Discussion

Upland cotton is a commonly cultivated fiber crop for the textile industry. Meeting the increasing food and fiber demands of the rising global population and addressing unpredictable climatic shifts requires enhancing crop productivity on the same or reduced land area, thus using the same or fewer resources. Combining higher-density planting with improved photosynthetic capacity is a promising approach to achieving this objective. This would maximize resource utilization while optimizing the plant’s energy capture and conversion efficiency resulting in higher crop yields with resource efficiency, thereby benefiting growers and consumers. Understanding the impact of plant architecture on cotton growth is paramount to enable the breeding of cotton varieties with enhanced architectural traits (Kaggwa-Asiimwe et al., 2013; Hollender et al., 2018).

The genetic modification of plant architecture can offer several advantages such as increased carbon assimilation, improved light utilization in dense crop canopies, heightened mechanical process efficiency, decreased susceptibility to insects and diseases by reducing canopy humidity, and the potential for higher yields. The growth and yield of cotton plants are significantly impacted by light penetration, especially in dense cotton fields where the upper parts of the plant receive most of the light. This uneven distribution of light within the branch canopy critically impacts optimal growth and yield. Light distribution is influenced by various factors, such as genetics, environment, and management practices (Mao et al., 2014; Yao et al., 2017). Cotton varieties with columnar canopies tend to have an open structure that facilitates better radiance interception and light penetration throughout the canopy (Chapepa et al., 2020). The positioning of bolls is also crucial for yield, with the first position bolls proportionally contributing to plant yield due to their larger size and weight. To produce these bolls, older leaves must produce enough photosynthates, which require even and adequate light penetration throughout the plant (Jiang et al., 2012; Hollender et al., 2018; Huang et al., 2022). Therefore, developing cotton cultivars with modified plant architecture can significantly improve crop performance and have far-reaching benefits for the cotton industry. Here, we evaluate the genomic content, phylogenetic distribution, and functional implications of *TAC1* in cotton.

Phylogenetic analysis of *TAC1* revealed between 1 and 2 copies in representative diploid Angiosperms, except for the diploid progenitors of the *Gossypium* lineage (*G. arboreum* and *G. raimondii*, which contained 3 copies, **Figure 1**; **Table 1**, indicating that the *TAC1* gene duplication is specific to this lineage. In *Prunus*, a species with a single *TAC1* copy, altered expression of this gene resulted in pleiotropic shoot phenotypes. For example, silencing of *TAC1* resulted in plum trees with severely vertical branch orientations, while overexpression resulted in trees with more horizontal branch orientations. Collectively, alteration of *TAC1* in *Prunus* species leads to pleiotropic shoot phenotypes (Dardick et al., 2013; Hollender et al., 2020). Similarly in *Arabidopsis* plants with a single copy of the *TAC1* gene, mutant plants display a more inclined branch angle relative to the wild type (Hollender et al., 2020). A study on the functional characterization of *TAC1* in *Populus trichocarpa* (poplar), which contains two copies of the gene (Potri.014G102600 and Potri.002G175300), revealed that knocking out these genes resulted in narrower leaf angles and upright shoot growth (Fladung, 2021). In *Malus domestica*, two members of the *TAC1* gene (*MdTAC1a* and *MdTAC1b*) have been identified. Subcellular localization analysis of *MdTAC1a* showed that it is detected in the nucleus and cell membrane, while *MdTAC1b* is detected only in the cell membrane. Both genes play a role in regulating branch inclination in *Malus domestica*, and they are highly expressed in the shoot tips and vegetative buds of weeping cultivars. However, they exhibit weak expression in columnar cultivars (Li et al., 2022).

Our results revealed three copies in the diploid progenitor cotton species (*G*. *arboreum* and *G*. *raimondii*) indicating the duplication event supersedes the polyploidization event in allotetraploid cotton and underscores the potential importance retaining this gene duplication event in success of both the wild and domesticated allotetraploid cottons (Cronn et al., 1999; Meng et al., 2020). Visual comparisons of cotton plants with various Angiosperms reveals that most cotton genotypes have near horizontal branches which could be attributed to the presence of additional *TAC1* alleles. However, certain naturally occurring mutant cotton varieties exhibit more acute branch angles compared to their wild relatives, which could be a result of gene regulation ((Ji et al., 2021). Moreover, previous studies have identified two types of cotton mutants, known as cluster branching (*cl1*) in *G. hirsutum* (Upland cotton) and short branch (*cl2*) in *G. barbadense* (Pima cotton), which exhibit a distinct phenotype called “*nulliplex-branch*” (*nb*). These mutants display determinate sympodial growth (Kearney, 1930; Pathak and Singh, 1975). The *cl1* mutant is associated with a recessive allele located on chromosome D07, while the *cl2* mutant is associated with a recessive allele located on chromosome A07 (Stephens, 1955; Endrizzi and Ray, 1992). The *nulliplex-branch* mutants demonstrate unique characteristics, such as flowers developing directly from leaf axils on the main stem or occasionally from a short branch with a single node. These phenotypes typically lack fruiting branches and possess a compact plant structure. As a result, they are highly suitable for high-density planting and mechanical harvesting. Additionally, they mature early, and their growth does not require chemical regulation or manual pruning, which reduces labor inputs (Silow, 1946; McGarry et al., 2016; Sun et al., 2016b; Si et al., 2018; Chen et al., 2019; Huang et al., 2022; Sun et al., 2022).

Sequence similarity analysis of a representative *TAC1* gene in cotton showed expected regions of high identity when compared to other species (cacao, peach, soybean, *Arabidopsis*, and maize), **Figure 2**. Protein family analysis of *TAC1* identified an NAD-Dependent protein deacetylase HST1-like domain (Panther 38366). This functional domain (histone deacetylation) is involved in telomeric silencing and methylation maintenance. Other studies have shown that LAZY and DRO interact with a protein that has an RCC1 (regulator of chromatin condensation) domain. The strong sequence similarity between the *TAC1* gene in Arabidopsis and the wild diploid cotton species *Gossypium raimondii* confirms the functional role of *TAC1* in wild cotton. This finding supports the notion that domestication processes resulted in targeted artificial selection for agronomic traits, including yield, pest resistance, fiber length, compact branching, and reduced growth and maturity periods. The multiple alignment of *TAC1* amino acid sequences (**Figure 2**) also showed structural similarities in the *IGT* domain, suggesting that these ancient IGT genes play a consistent role in determining shoot growth angle orientation (Dardick et al., 2013; Dong et al., 2016; Montesinos et al., 2021).

An analysis of the six homeologous copies of *TAC1* in the Coker312 genotype revealed intriguing differences in the level of homeologous and homologous similarity among the six *TAC1* copies at the sequence identity level. Notably, the A11/D11 copies of *TAC1* exhibited a 96.8% identity, while their similarity with homologs on chromosome 8 or 12 was only up to 58% (**Supplementary Table 1**). We observed that this pair of *TAC1* homologs displayed dominant tissue-specific expression when compared to the other homologs. Additionally, their expression patterns displayed subgenome bias as shown in **Figures 3A, B**, **4A**. The A08/D08 and A12/D12 copies exhibited a sequence identity of approximately 74% between homeologs, respectively (refer to **Supplementary Table 1** for details). Significantly, the expression patterns of A11 and A12 *TAC1* homologs in various vegetative tissues, including leaf, stem, meristem, and root, revealed a distinct prevalence of expression in leaf and stem tissues (**Figure 4**). Previous studies have demonstrated that *TAC1* exhibits increased expression in actively growing vegetative buds, despite its low expression levels in meristem tissue (Xu et al., 2017). The low expression of *TAC1* in root tissue aligns with its regulatory role in upper branches of plants, with minimal or no expression in lower branches (Dardick et al., 2013). Our analysis showed a notable difference in the expression levels of A11 and D11 *TAC1* copies, with A11 exhibiting significantly high expression in flower tissue and D11 displaying the highest expression in fiber tissue. These results highlight the subgenome expression bias and suggest that the functional domains of the A11/D11 coding sequence (CDS) may be redundant, while their regulatory regions (5’ and 3’ UTRs and promoters) could be differentially activated in various tissues and developmental stages (**Figure 4**). Previous studies have indicated that *TAC1* exhibits high expression in reproductive structures such as flower buds, which may explain the horizontal growth of the flower and its subsequent development into fiber (Dardick et al., 2013) The A12/D12 copies displayed higher expression levels in vegetative tissues, with the A12 copy showing subgenome expression bias over D12 in leaf, stem, meristem, and root tissues, indicating the possibility of functional redundancy or sub-functionalization with a role in leaf angle.

Targeted knockout of the A11/D11 homeologous *TAC1* copies resulted in a cotton plant with an induced columnar phenotype with distinct branch and petiole inclination, **Figure 6**; **Supplementary Figure S5** and S6. Overall, the mutant plants consistently exhibited narrower measurements in terms of branch inclination angle and petiole angle for sympodial branches. However, there were no notable differences observed in the measurements of monopodial branches and petiole angle for both the mutant and wild type Coker 312 (**Figures 6B, C**). No variations were observed in some morphological traits, including branch length (monopodial and sympodial branches), plant height, and boll count (**Table 6**). Sequence analysis of the mutants from three independent events with columnar branching (*tac1-72*, *tac1-73*, and *tac1-74*) revealed a high efficiency of the CRISPR-induced mutations (**Figure 5A, B**) resulting in hemizygous/homozygote mutations with large deletions and a fragment rearrangement (**Supplementary Tables S6**, **S7**). In *tac1-72* and *tac1-73* mutants (**Figure 5A**) a 94bp deletion was observed in both A11/D11 homeologs. Interestingly, the analysis of *tac1-74* mutant revealed an 89bp inversion flanked by two deletions at the target site in D11 and the 94bp deletion in A11 (**Figure 6B**). These mutations altered the *GhTAC1* gene function, rendering it non-functional, and subsequently led to the same observed columnar phenotype in all independent events. These findings align with previous studies in other Eudicots which have shown a correlation between mutations in *TAC*1 and the development of a nearly erect plant architecture phenotype (Dardick et al., 2013; Hollender et al., 2018; Hollender et al., 2020; Fladung, 2021; Dutt et al., 2022; Li et al., 2022). Intriguingly, we noted an enhanced canopy structure in the mutants accompanied by a significant increase in the number of leaves, auxiliary buds, and subtending leaves, particularly in the basal region of the plants compared to the apical region (see **Supplementary Figures S5**, **S6**). This pattern is characteristic of columnar plant phenotypes which have the potential to improve light distribution within the plant canopy (Chapepa et al., 2020). Validation of the expression of both A11/D11 homeologs in stem tissue revealed a decrease in expression levels for A11/D11 pairs. This decline in expression for both gene pairs contributes to the columnar phenotype observed in the mutants. These findings are consistent with earlier research which indicates that the loss/mutation of *TAC1* gene function results in decreased expression in mutant varieties (Dardick et al., 2013; Hollender et al., 2020).

The segregation analysis of T_1_ progeny derived from the mutants, particularly *tac1-73* and *tac1-74*, revealed a notably favorable segregation ratio, emphasizing a single copy and a confirmation supported by the presence of resistance to the bar gene selectable marker (**Supplementary Figures S5**, **S6**). These findings provide strong evidence of transformation efficiency.

Several studies have indicated that the canopy microenvironment plays a critical role in regulating not only the distribution of light but also the temperature and relative humidity within the canopy. These factors collectively influence the radiation received by plants (Yang et al., 2014; Chapepa et al., 2020). The findings of this study are consistent with previous studies in peach and poplar, where mutant varieties with loss of function of the *TAC1* gene showed pronounced nearly upright growth of branches (Dardick et al., 2013; Fladung, 2021). The potential benefits of upright branch growth in peach, poplar, and other woody Eudicots include high density planting, increased yield, automated harvesting, and reduced pruning (Dardick et al., 2013; Fladung, 2021). Harnessing knowledge of plant architecture offers the potential for ultimately enhancing cotton productivity and increasing higher yields.

## Conclusion

Plant architecture undergoes dynamic changes throughout their development. Our study revealed that the *TAC1* gene is duplicated and unique to the Gossypium lineage within the Angiosperms. Interestingly, we observed a correlation between the number of native *TAC1* copies and the horizontal branching pattern, with cotton exhibiting the most pronounced lateral growth among the Angiosperms. We also confirmed CRISPR-mediated knockout of the highly expressed copies of the *TAC*1 gene (A11G109300 and D11G112200) led to a significant reduction in both branch and petiole angles. The results of this study demonstrate the potential of gene editing technology to introduce novel traits into high-value crops, such as cotton, that would otherwise not exist naturally because of the presence of multiple subgenomes. These findings have significant implications for improving planting density, understanding optimal light interception and the correlation with yield potential, and enhancing our understanding of the evolution and domestication of the *Gossypium* lineage.

## Data availability statement

The original contributions presented in the study are included in the article/**Supplementary Material**. Further inquiries can be directed to the corresponding author.

## Author contributions

FK: Investigation, Writing – original draft, Methodology. SK: Methodology, Writing – original draft, Supervision, Writing – review & editing. ZL: Supervision, Writing – review & editing, Data curation, Formal Analysis. AS: Data curation, Formal Analysis, Writing – review & editing. CD: Conceptualization, Formal Analysis, Writing – original draft, Writing – review & editing. DJ: Resources, Writing – review & editing. CS: Conceptualization, Funding acquisition, Investigation, Project administration, Resources, Supervision, Writing – original draft, Writing – review & editing.

## Supplementary material

Supplementary Figure 1 Dual guide RNA design

Supplementary Figure 2 The total DNA amplified using primer pair: F-AGATGGGCTTGCACGAAATGTTAAG and R-CGTTTTTGGCAGGAAGAGRAGATG. The PCR products were cloned into pGEM-T-Easy vector and sequenced using standard Sanger sequencing techniques. The sequence alignments were conducted by Geneious Prime. For alignment, the sequences of A11G109300 and D11G112200 are from Coker312 whole genome sequence, the sequences of A1_Sp6 to A18_Sp6 are from sanger sequencing results. The 95-bp deletion showed in Clone A3-A4, the 95-bp deletion showed in Clone A1, A2, clone A6, A7, clone A10-A12, A14-A16 and A18; the one bp insertion (A) found in Clone A8 and A13; the 89-bp inversion detected in Clone A9 and A17. The highlighted line arrow indicated SNP between A11G109300 and D11G112200.

Supplementary Figure 3 A cartoon image describing the branch and petiole angle was measured in the study. In measuring branch angles, a line was drawn between the branch point and 2cm down the branch, and the angle with respect to the line and stem was recorded. For petiole angle measurements, a line was drawn tangent to the branch and 1cm back from the last petiole on the branch, and the angle of this line with respect to the angle of the stem was recorded (Adapted from "Cotton Plant", Adapted from "Cotton Plant" by BioRender.com (2023). Retrieved from https://app.biorender.com/biorender-templates.

Supplementary Figure 4 Branch and leaf count at the apical part (at 45cm height)

Supplementary Figure 5 Images of mutant plants (*tac1-72*, *tac1-73*, and *tac1-74*)

Supplementary Figure 6 Representative images illustrating the comparison between Wild Type Coker 312 and T_1_ generation of mutant plants (*tac1-73* and *tac1-74*) at vegetative growth (before squaring).
