# Supplementary figures and images for: Phylogenetic and functional analysis of tiller angle control homeologs in allotetraploid cotton
Source: Front Plant Sci. 2024 Jan 31;14:1320638. doi: 10.3389/fpls.2023.1320638 (PMC10864623; doi:10.3389/fpls.2023.1320638)

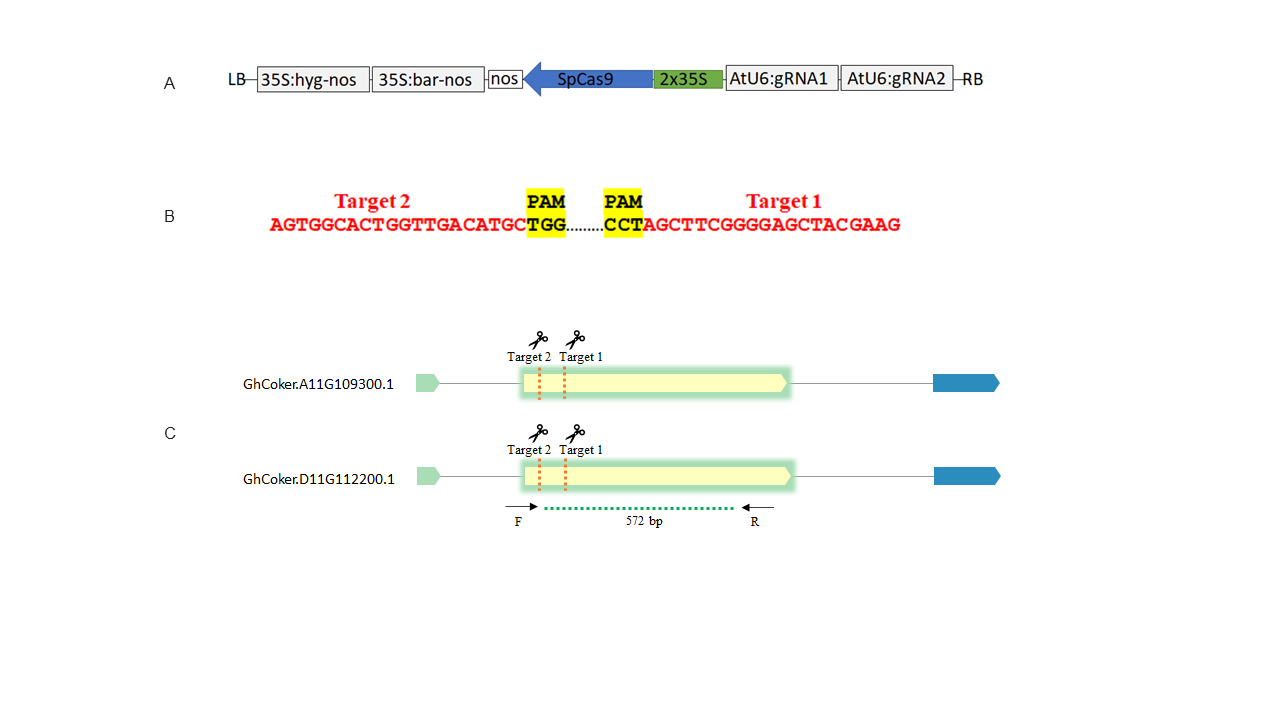

Supplement: Supplementary Figure 1 — Dual guide RNA design [file Image_1.tif]

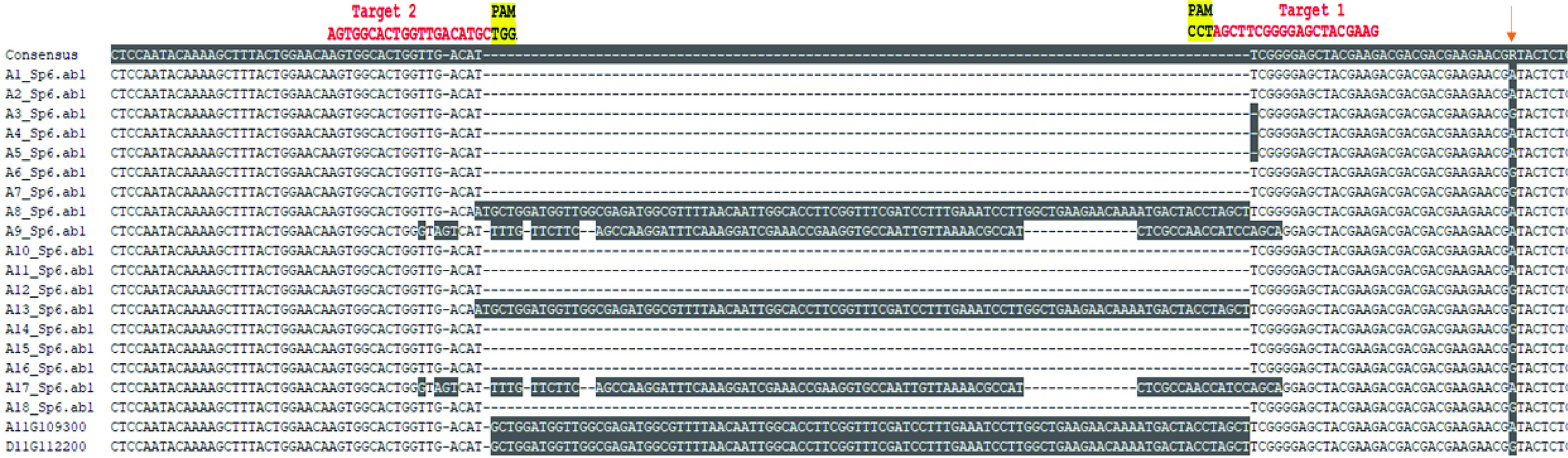

Supplement: Supplementary Figure 2 — The total DNA amplified using primer pair: F-AGATGGGCTTGCACGAAATGTTAAG and R-CGTTTTTGGCAGGAAGAGRAGATG. The PCR products were cloned into pGEM-T-Easy vector and sequenced using standard Sanger sequencing techniques. The sequence alignments were conducted by Geneious Prime. For alignment, the sequences of A11G109300 and D11G112200 are from Coker312 whole genome sequence, the sequences of A1_Sp6 to A18_Sp6 are from sanger sequencing results. The 95-bp deletion showed in Clone A3-A4, the 95-bp deletion showed in Clone A1, A2, clone A6, A7, clone A10-A12, A14-A16 and A18; the one bp insertion (A) found in Clone A8 and A13; the 89-bp inversion detected in Clone A9 and A17. The highlighted line arrow indicated SNP between A11G109300 and D11G112200. [file Image_2.tif]

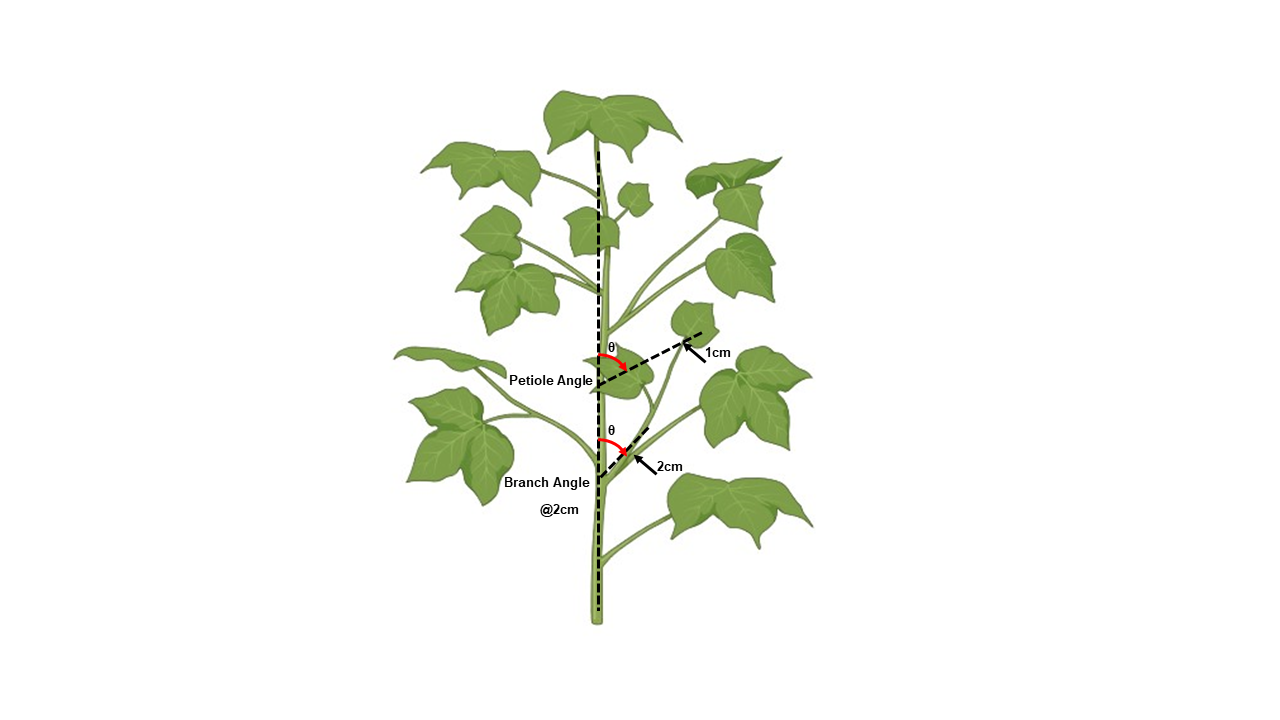

Supplement: Supplementary Figure 3 — A cartoon image describing the branch and petiole angle was measured in the study. In measuring branch angles, a line was drawn between the branch point and 2cm down the branch, and the angle with respect to the line and stem was recorded. For petiole angle measurements, a line was drawn tangent to the branch and 1cm back from the last petiole on the branch, and the angle of this line with respect to the angle of the stem was recorded (Adapted from "Cotton Plant", Adapted from "Cotton Plant" by BioRender.com (2023). Retrieved from https://app.biorender.com/biorender-templates. [file Image_3.tif]

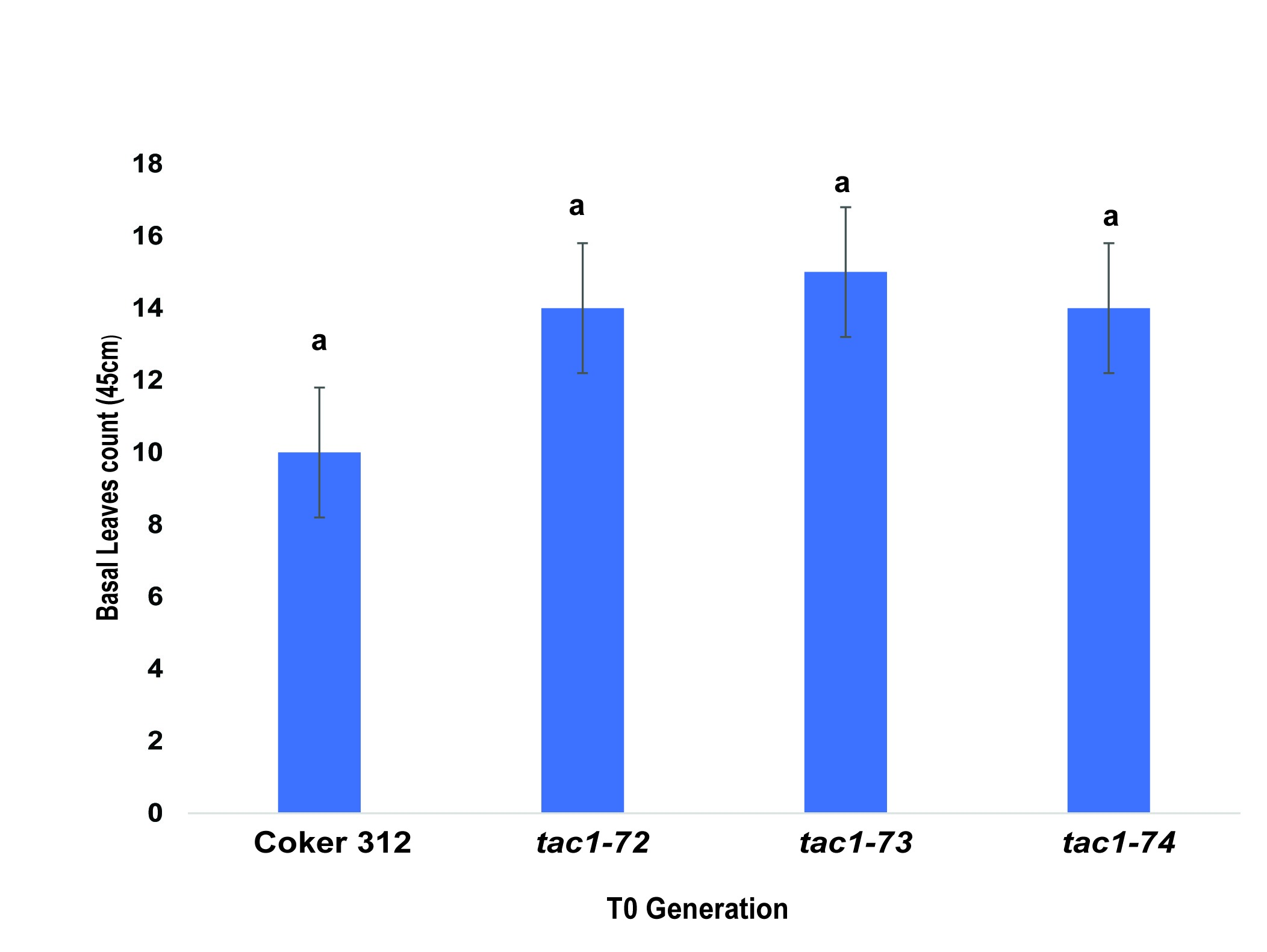

Supplement: Supplementary Figure 4 — Branch and leaf count at the apical part (at 45cm height) [file Image_4.tif]

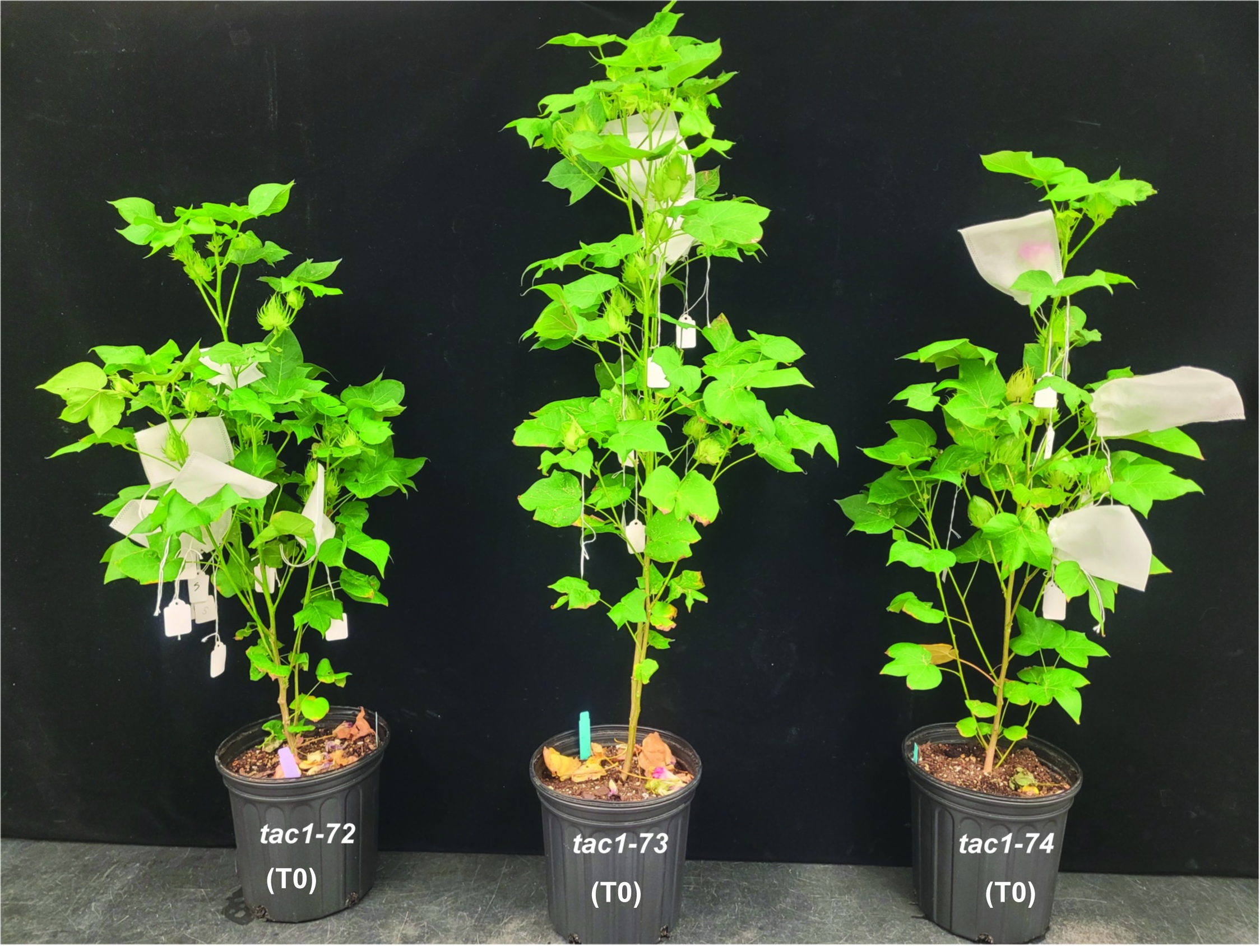

Supplement: Supplementary Figure 5 — Images of mutant plants (tac1-72, tac1-73, and tac1-74) [file Image_5.tif]

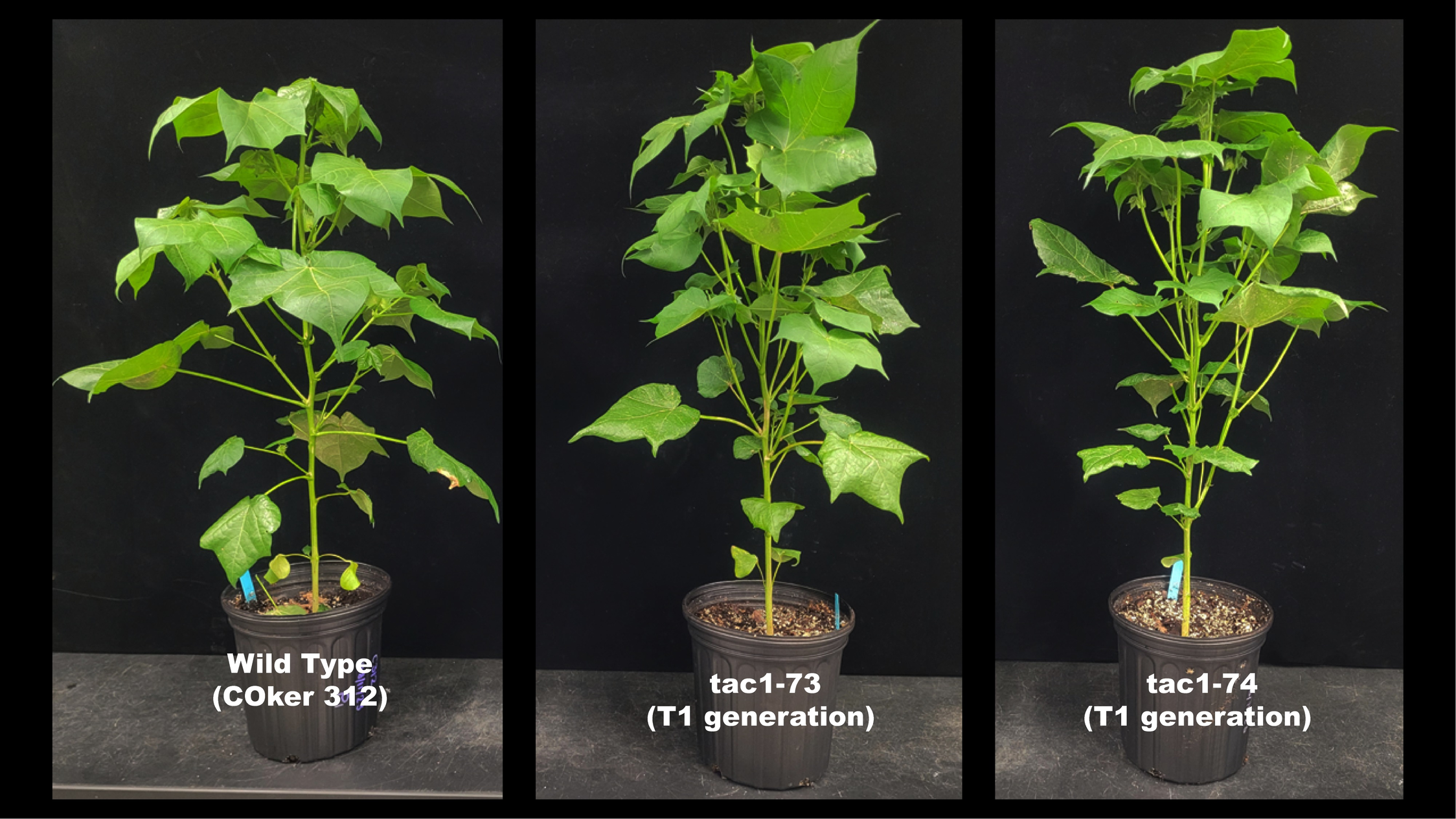

Supplement: Supplementary Figure 6 — Representative images illustrating the comparison between Wild Type Coker 312 and T1 generation of mutant plants (tac1-73 and tac1-74) at vegetative growth (before squaring). [file Image_6.tif]
